# Evaluation of Self-Collected Versus Health Care Professional (HCP)-Performed Sampling and the Potential Impact on the Diagnostic Results of Asymptomatic Sexually Transmitted Infections (STIs) in High-Risk Individuals

**DOI:** 10.3390/idr15050047

**Published:** 2023-08-25

**Authors:** Simon Weidlich, Sven Schellberg, Stefan Scholten, Jochen Schneider, Marcel Lee, Kathrin Rothe, Nina Wantia, Christoph D. Spinner, Sebastian Noe

**Affiliations:** 1 Department of Internal Medicine II, School of Medicine, University Hospital Rechts der Isar, Technical University of Munich, Ismaninger Str. 22, 81675 Munich, Germany; jochen.schneider@mri.tum.de (J.S.);; 2 Novopraxis Berlin, Mohrenstr 6, 10117 Berlin, Germany; 3 Praxis Hohenstaufenring Köln, Richard-Wagner-Str 9-11, 50674 Cologne, Germany; 4 Institute for Microbiology, Immunology and Hygiene, School of Medicine, Technical University of Munich, Trogerstr. 30, 81675 Munich, Germany; 5 German Center for Infection Research (DZIF), Partner Site Munich, 80802 Munich, Germany; 6 MVZ München am Goetheplatz, Waltherstr. 32, 80807 Munich, Germany

**Keywords:** sexually transmitted infection (STI), *Chlamydia trachomatis* (CT) infection, *Neisseria gonorrhoeae* (NG), self-sampling, healthcare professional guided sampling, men having sex with men (MSM)

## Abstract

Sexually transmitted infections (STIs) are increasing among men who have sex with men (MSM). Screening can improve the detection and outcome of asymptomatic STIs in high-risk populations. Self-sampling may be a resource-optimized strategy; however, its diagnostic reliability compared to testing by healthcare professionals (HCPs) requires further investigation. In this prospective, multicenter cohort study in a high-income country, asymptomatic MSM with a sexual risk profile for STIs were included. Sequential swabs for STI nucleic acid-based diagnosis of *Chlamydia trachomatis* (CT) and *Neisseria gonorrhoeae* (NG) were performed after randomization, either through self-sampling or HCP-performed sampling. Baseline demographic information, sexual risk behavior, and acceptance and feedback on self-sampling were recorded using an electronic questionnaire. Out of 236 asymptomatic MSM, 47 individuals (19.9%) tested positive for CT and/or NG through self- or HCP-performed sampling. For CT, the sensitivity was 93.3% for both sampling methods, while for NG, it was 90.0% for self-sampling and 95.0% for HCP-performed sampling. Our study demonstrates that self-sampling for asymptomatic STIs has a comparable diagnostic outcome to HCP-performed sampling, with high acceptance in high-risk MSM.

## 1. Introduction

Sexually transmitted infections (STIs) are increasing worldwide, particularly among men who have sex with men (MSM). According to the World Health Organization (WHO), there are approximately 350 million new cases of bacterial STIs reported worldwide each year [1]. High rates of STIs have been reported for MSM both by the Centers for Disease Control, the European Centre for Disease Control, and in national reports from Germany [2,3,4,5,6,7,8]. In a previous study, our research group published data on the prevalence of STIs in sexually active, asymptomatic MSM in Germany. This study revealed that 13.5% of individuals had positive samples for either *Neisseria gonorrhoeae* (NG) and/or *Chlamydia trachomatis* (CT) among subjects living with HIV (PLWH) [9]. This is in line with recently published data indicating that only 31.1% of STI-infected MSM reported STI-related symptoms [10,11]. This suggests the potential additive value of STI screening in individuals at increased risk for STIs [12].

The current German national guideline for the use of HIV pre-exposure prophylaxis (HIV-PrEP) in individuals with increased risk of HIV acquisition and European guidelines both recommend regular screening for STIs [13,14]. Despite these guidelines, the implementation of regular STI screening and testing is challenging for several reasons, even in high-income countries. These include, but are not limited to, the restricted availability of healthcare professionals (HCPs), inconvenience of sampling (such as with urethral swabs) methods, and time constraints. To address these challenges, self-sampling has emerged as one potential solution.

Recently, several studies on the self-sampling of NG and CT have been published; however, few have focused on asymptomatic STIs in MSM [15]. Although self-sampling might be a reliable alternative to HCP-performed sampling, a recently published review of 45 studies in a very heterogeneous population found that only two were randomized studies [16]. Therefore, this study aims to add evidence to the diagnostic reliability of two novel certificated self-collection devices in comparison to HCP-performed sampling for CT/NG diagnostics. We hypothesized that self-sampling will demonstrate comparable sensitivity to HCP-performed sampling, as measured using the proportion of positive samples for the investigated STIs in both methods. Additionally, we sought to further investigate the acceptance of self-sampling by collecting information on anticipated user-reported challenges during self-sampling and identifying new challenges encountered by participating individuals. Furthermore, this study also aimed to gauge the extent to which at-risk individuals accept self-sampling as an alternative and understand the underlying reasons for non-acceptance.

## 2. Materials and Methods

### 2.1. Study Population and Procedures

Individuals self-identifying as MSM and reporting >2 condomless anal intercourses with ≥2 male sex partners within the last 24 weeks prior to their inclusion in the study were recruited from April 2021 to July 2022. These individuals were recruited during regular sexual health check-up visits at one of the three participating German medical centers in Berlin, Cologne, and Munich. The local ethics committee approved this study, and written informed consent was obtained from every participant before inclusion in the study.

Symptomatic MSM with suspicion of an active STI or reporting a history of a recent diagnosis of an STI (within 14 days prior to inclusion in the study) were not considered for inclusion. All participants received instructions on the proper use of swabs through pictograms accompanied by explanatory text (see  Supplementary Figures S1–S4). They were required to self-sample oropharyngeal and anal swabs using the Copan FLOQSwab 552C^®^* (Copan, Brescia, Italy) and collected urine samples using the Copan Self-UriSponge system^TM^ 8E031S100* (Copan, Brescia, Italy), which was specifically designed for self-sampling and previously validated [17,18]. Transport Medium 900-0601 (Copan, Brescia, Italy) was immediately added to dry swabs for analysis by HCPs. Additionally, rectal and oropharyngeal samples (using eSwab^®^ 490CE02), as well as urethral samples (using eSwab^®^ 483CE (both Copan, Brescia, Italy) that have already been established for standard use were collected by HCPs.

Dry swabs were chosen for self-sampling to prevent any spillage of the liquid transport medium by the enrolled individuals. Real-time PCR for CT/NG was performed immediately using the same Cepheid Xpert CT/NG assay and software Dx system version 4.8 (Cepheid AB, Solna, Sweden) for all samples. All positive STI results were included in the analysis. The order of sampling (self-sampling first versus HCP-performed sampling first) was randomized at a 1:1 ratio using a paper envelope system. In addition to the samples, all individuals were asked to complete a standardized electronic questionnaire to assess their individual risk profiles and acceptance of self-sampling.

### 2.2. Statistical Analysis

According to published data, a 20% prevalence of STIs for CT/NG was assumed, with an estimated 7% of discordant pairs based on previous findings [19]. Using McNemar’s test for paired samples with α = 0.05 and β = 0.20 resulted in an estimated total sample size of 218 subjects. Considering a 10% rate of invalid test results for reasons other than inadequate sampling, the overall sample size was increased to 240 subjects. Therefore, 80 individuals per center were planned for enrollment at the three participating centers.

For descriptive statistics, medians with the 25th and 75th quantiles or absolute numbers with ratios or percentages were calculated. Sensitivity was calculated as the ratio of positive samples identified using the method of interest to the number of subjects testing positive using either of the two sampling methods for the specific areas of interest. Additionally, 95% confidence intervals for the sensitivities were calculated using the method described by Clopper and Pearson for binomial distributions without further adjustment for prevalence variability.

## 3. Results

Overall, 236 individuals were enrolled in the study, and all were suitable for analysis. The baseline characteristics of the overall study sample, as well as the samples per study site, are displayed in Table 1. A total of 47 (19.9%) individuals tested positive for CT and/or NG using either self- or HCP-performed sampling. The distribution of individuals testing positive in at least one obtained sample was similar across the study sites, with 13 (16.2%), 17 (21.5%), and 17 (22.1%) in Berlin, Cologne, and Munich, respectively. Among all swabs, 30 (12.7%) individuals tested positive for CT, 20 (8.5%) tested positive for NG, and 3 (1.3%) had confirmed simultaneous CT and NG infections.

Regarding CT detection, out of the 30 individuals who tested positive using either sampling method, 28 were correctly identified via both self-sampling and HCP-performed sampling, resulting in a sensitivity estimate of 93.3% (CI: 77.9; 99.2) for both sampling methods. For NG detection, out of the 20 individuals who tested positive using either sampling method, 18 and 19 were correctly identified by self-sampling and HCP-performed sampling, respectively. This yielded sensitivity estimates of 90.0% (CI 68.3; 98.8) and 95.0% (CI 75.1; 99.9) for self-sampling and HCP-performed sampling, respectively. Sensitivities and specificities for each oropharyngeal, rectal and urethral swab are listed in Table 2. Additional details on samples that were only positive for CT or NG in one of the sampling methods are displayed in Table 3.

The order of sampling (self-sampling or HCP-performed sampling first) did not significantly affect the diagnostic outcomes. No differences in STI detection were observed between PLWH- and HIV-seronegative individuals. Detailed results for swabs obtained from different locations and per sampling method are displayed in Table 2. Invalid diagnostic results were observed in two (0.8%) and twelve (5.1%) rectal samples (*p* < 0.001), four (1.7%) and eight (3.4%) oropharyngeal samples (*p* = 0.067), and four (1.7%) and fourteen (1.7%) urine/urethral samples (*p* = 1.000) collected via self- or HCP-performed sampling, respectively.

**Figure 1:**
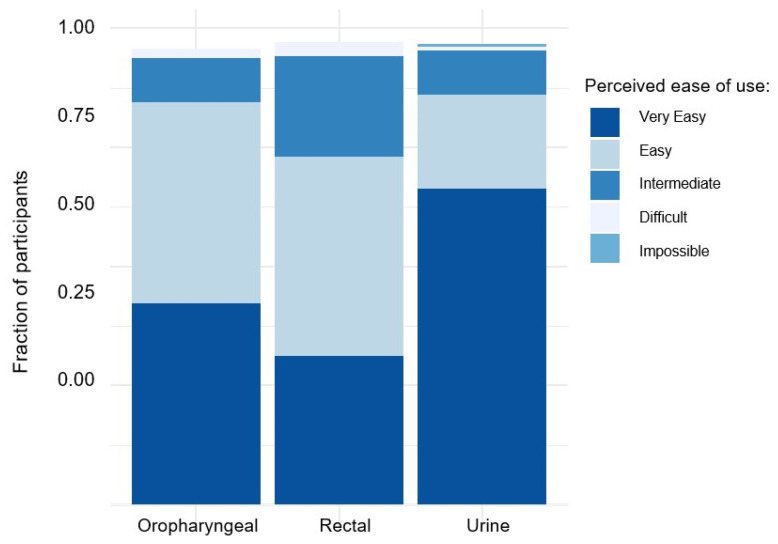
Perceived ease of use for self-sampling of rectal, oral, and urine samples among study participants.

For rectal, oropharyngeal, and urine sampling, 173 (75.2%), 200 (88.1%), and 204 (89.1%) respondents found the procedures for self-sampling to be “very easy” or “easy” (Figure 1). The responses to whether subjects encountered several anticipated challenges during the rectal, oropharyngeal, and urine sampling procedures can be found in Supplementary Tables S1–S6. Among 207 individuals responding to whether they could imagine performing self-sampling in the future, 187 (90.3%) and 20 (9.7%) answered “yes” and “no”, respectively. Only a small number of individuals, specifically nine respondents, self-reported ‘negative’ attitudes towards self-sampling. These negative attitudes included perceiving self-sampling as too complicated (*n* = 5) or too time-consuming (*n* = 2), concerns regarding whether results obtained from self-sampling would be less reliable (*n* = 1), and unexplained preferences towards HPC-performed sampling (*n* = 1).

## 4. Discussion

In this study, we compared self-sampling and HCP-performed sampling in a group of MSM at high risk for STIs. Within the assessed cohort, we observed a comparable frequency for asymptomatic STIs, as seen in previously published study data. Our data demonstrated comparable estimates for the sensitivity of rectal and oropharyngeal CT and NG PCR diagnostics after self-sampling with Copan FLOQSwab and HCP-performed sampling with the Copan eSwab^®^ system. In addition, comparable sensitivities for CT and NG PCR diagnosis in self-collected urine using the Copan Self-UriSponge system and in HCP-performed urethral eSwabs were observed. These findings support the notion that self-sampling is equally reliable as HCP-performed sampling in the assessed population. This is in line with previously published clinical studies [16,19].

Of note, we considered results from the two sampling techniques to be equally valuable and, in particular, not to have different probabilities in terms of ‘false positives’. Therefore, in case of discordant results, the ‘positive’ test was considered ‘true’. We chose this approach in order to avoid biased results as a result of preferring any of the two sampling techniques as the reference. However, it must be noted that this approach might have led to an overestimation of the proportion of people with STIs.

Overall, our study revealed only a few discordant results, affecting only seven (3.0%) of the enrolled individuals. These discrepancies were primarily attributed to invalid test results in either sampling method. However, the occurrence of invalid tests was lower than initially assumed for statistical calculations and fell within the clinically observed frequency. The number of invalid tests was significantly higher when performed by a HCP, especially for rectal sampling. This could be because sampling might be performed more tentatively when performed on another individual than when performed on oneself, leading to higher amounts of invalid tests. Notably, we observed instances where samples tested positive in HCP-performed sampling but not in self-sampling, and vice versa. Therefore, self-sampling does not seem to introduce a systematic bias towards a lower detection rate of STIs or more invalid tests per se.

In addition to demonstrating a high degree of reliability, our data also indicate that the acceptance rate of self-sampling methods is high. Most participants in this study reported the procedure to be “very easy” or “easy” for each sampling location. Notably, urine sampling showed particularly high acceptance. This finding is significant, considering the earlier description of convenience as a crucial factor in implementing widespread STI self-testing [12]. Based on clinical experience, fear of discomfort associated with urethral swabs discourages regular testing for urethral STIs, and in our study, results from urethral swabs from HCPs and urine samples from self-sampling led to identical results. It should be mentioned at this point that acceptance for self-sampling might be higher in our cohort than in the general population who might not be seeking sexual health services on a regular basis. However, for these people, self-sampling might be even more convenient than speaking to an HCP.

Despite the positive outcomes, we also identified some challenges that need to be addressed. Most importantly, the use of dry swabs proved to be challenging for anal self-sampling, as it not only complicated the insertion of the swab but also caused pain (18.7%) and even led to bleeding (Supplementary Table S1). To improve handling and acceptance, an alternative approach could involve the use of moistened liquid medium swabs. Additionally, correct handling of anal swabs proved to be a frequently raised concern. Problems with the proper execution of anal swabs may have been a major reason for invalid tests in self-collected samples. Providing more precise instructions and encouraging regular practice could be key to enhancing the efficacy of testing. One methodological objective could be the use of different swab systems for self-sampling and HCP-performed sampling. However, the detection rates were similar for both swab systems (eSwab^®^ and FLOQSwabs^®^, Copan, Brescia, Italy).

It should be noted that this study was not specifically designed to assess diagnostic accuracy for each sampling location (rectal, oropharyngeal, and urethral) due to low incidences of asymptomatic STIs. A much larger number of participants would have been required to achieve sufficient statistical power. However, when considering all sampling locations collectively, our trial demonstrates that self-sampling has a similar sensitivity to HCP-performed sampling.

In conclusion, self-sampling has both a comparable diagnostic outcome to HCP-performed sampling and a high rate of user acceptance. Therefore, it should be used in clinical practice to encourage regular STI testing.

## Figures and Tables

**Table 1:** Baseline characteristics of subjects enrolled in this study (results per site).

	Total	Berlin	Cologne	Munich
Subjects enrolled in the study	236	80	79	77
Age [years]	37	37	39	36.5
(Q1; Q3)	(31; 44.75)	(31; 42)	(31; 47)	(32; 43)
Missing values, n (%)	26 (11.0)			
Born in Germany, n	174	63	60	51
(%)	(76.0)	(80.8)	(76.9)	(65.4)
Missing values, n (%)	7 (3.0)			
Living with HIV, n	44	7	28	9
(%)	(18.8)	(8.8)	(35.0)	(11.2)
Missing values, n (%)	2 (0.8)			
Identifying as MSM, n	207	69	72	66
(%)	(89.6)	(87.3)	(91.1)	(83.5)
Missing values, n (%)	5 (2.1)			
Having more than 2 sex partners in the last 3 months, n	181	60	62	59
(%)	(76.7)	(75.0)	(77.5)	(73.8)
Missing values, n (%)	0 (0.0)			

Abbreviations: MSM: men who have sex with men.

**Table 2:** Results of reactive swabs and urine samples for self- and healthcare professional (HCP)-performed sampling per location and infection.

	Reference	Self-Sampling			HCP-Sampling		
	Positive in Either Sample	n	Sensitivity	Specifity	n	Sensitivity	Specifity
*C. trachomatis*							
Oropharyngeal swab	5	4	0.80 (0.28; 0.99)	0.97 (0.94; 0.99)	3	0.60 (0.15; 0.95)	0.99 (0.97; 1.00)
Rectal swab	23	22	0.96 (0.78; 1.00)	0.86 (0.81; 0.90)	21	0.91 (0.72; 0.99)	0.90 (0.86; 0.94)
Urethral swab/urine	5	5	1.00 (0.48; 1.00)	0.96 (0.93; 0.98)	5	1.00 (0.48; 1.00)	0.96 (0.93; 0.98)
*N. gonorrhoeae*							
Oropharyngeal swab	13	9	0.69 (0.39; 0.91)	0.98 (0.94; 1.00)	12	0.92 (0.64; 1.00)	0.98 (0.96; 1.00)
Rectal swab	13	13	1.00 (0.75; 1.00)	0.95 (0.91; 0.98)	13	1.00 (0.75; 1.00)	1.00 (0.98; 1.00)
Urethral swab/urine	2	2	1.00 (0.16; 1.00)	0.98 (0.96; 1.00)	2	1.00 (0.16; 1.00)	0.98 (0.96; 1.00)

**Table 3:** Further details for samples exclusively positive in one sampling method.

***C. trachomatis***		
*Positive in self- and HCP-performed sampling (n)*	*Only positive in self-sampling (n)*	*Only positive in HCP-performed-sampling (n)*
30	2	2
	1 positive oropharyngeal and anal swab in self-testing, both tests negative when tested by HCPs(HCP-performed sampling first)	1 positive anal swab in HCP-performed testing and an invalid swab when using self-sampling (HCP-performed sampling first)
	1 positive oropharyngeal and anal swab in self-testing, invalid anal swab, negative oropharyngeal swab (HCP-performed sampling first)	1 positive oral swab by the HCP and a negative swab from self-sampling (self-sampling first)
***N. gonorrhoeae***		
*Positive in self- and HCP performed sampling (n)*	*Only positive in self-sampling (n)*	*Only positive in HCP-performed-sampling (n)*
20	1	2
	1 positive oropharyngeal swab (HCP-performed sampling first), but CT positive in both sampling methods	1 positive oropharyngeal swab in HCP-performed sampling, but invalid in self-sampling (self-sampling first)
		1 positive oropharyngeal swab in HCP-performed sampling, but negative in self-sampling (self-sampling first)

Abbreviations: Health care professional (HCP), *Chlamydia trachomatis* (CT), *Neisseriae gonorrhoeae* (NG).

## Data Availability

Data can be made available by the corresponding author upon reasonable request.

## Supplementary Materials

The following supporting information can be downloaded at: https://www.mdpi.com/article/10.3390/idr15050047/s1, Figure S1: General study information, Figure S2: Manual for oropharyngeal sampling, Figure S3: Manual for rectal sampling, Figure S4: Manual for urine collection; Table S1: Challenges reported by participants in rectal self-sampling, Table S2: Additional free-text answers concerning additional challenges/problems reported for rectal self-sampling. Only answers occurring more than once are listed, Table S3: Challenges reported by participants in oropharyngeal sampling, Table S4: Additional free text answers concerning additional challenges/problems reported for oropharyngeal self-sampling. Only answers occurring more than once are listed, Table S5: Challenges reported by participants in urine self-sampling, Table S6: Additional free text answers concerning additional challenges/problems reported for urine sampling. Only answers occurring more than once are listed.
