# Supplementary material for: Evaluation of Self-Collected Versus Health Care Professional (HCP)-Performed Sampling and the Potential Impact on the Diagnostic Results of Asymptomatic Sexually Transmitted Infections (STIs) in High-Risk Individuals
Source: Infect Dis Rep. 2023 Aug 25;15(5):470–7. doi: 10.3390/idr15050047 (PMC10514875; doi:10.3390/idr15050047)
Supplement: Supplementary file 1 [file idr-15-00047-s001.zip › idr-2557990-supplementary.pdf]

Supplementary Material

Table S1: Challenges reported by participants in rectal self-sampling.

	n	%
Pain	43	18.7
Bleeding	2	0.9
Problems inserting the swab	1	0.4
Problems transferring the swab to the container	2	0.9
I did not understand the written instructions on how to perform the swab	4	1.7
I needed help from another person	0	0.0

Table S2: Additional free-text answers concerning additional challenges/problems reported for rectal self-sampling. Only answers occurring more than once are listed.

	n
Dryness	10
Difficulty identifying orifice	4
None	6
Swab depth challenges	3
Swab insertion challenges	3

Table S3: Challenges reported by participants in oropharyngeal sampling.

	n	%
Pain	14	6.1
Bleeding	0	0.0
Gagging	0	0.0
Problems transferring the swab to the container	0	0.0
I did not understand the written instructions on how to perform the swab	1	0.4
I needed help from another person	0	0.0

Table S4: Additional free text answers concerning additional challenges/problems reported for oropharyngeal self-sampling. Only answers occurring more than once are listed.

	n
Color coding challenges	2
Gagging	21
None	5

Table S5: Challenges reported by participants in urine self-sampling.

	n	%
Could not collect urine into the container	5	2.2
I did not understand the written instructions on how to perform the swab	11	4.8
I needed help from another person	0	0.0

Table S6: Additional free text answers concerning additional challenges/problems reported for urine sampling. Only answers occurring more than once are listed.

	n
Difficulties urinating	4
Missing information on how to proceed with the container	9
None	8

Figure S1. General study information.

Allgemeine Informationen zur SWAPTI-Studie

Vielen Dank für die Teilnahme an dieser Studie. Sie untersucht die diagnostische Genauigkeit von selbst entnommenen Abstrichen im Vergleich zu ärztlich entnommenen Abstrichen in der Diagnostik sexuell übertragbarer Erkrankungen.

Anleitung:

1. Nehmen Sie sich **genügend Zeit** für die Durchführung der Proben.
2. Wichtig ist es, **2-4 Stunden** vor der Uringewinnung keinen Urin gelöst zu haben.
3. Lesen Sie sich zunächst **alle Anleitungen** durch, um die Durchführung zu verstehen.
4. Führen Sie führen Sie **folgende Abstriche** durch:
   - → Rachenabstrich
   - → Uringewinnung
   - → Rektaler Abstrich
5. Geben Sie **die Proben an das Studienzentrum zurück**.
6. Bitte **beantworten** Sie uns noch **einige Fragen zum Ablauf** auf einem Tablet. Die Fragen und Antworten sind **pseudonymisiert** und die Mitarbeiter haben keinen direkten Zugriff auf Ihre eingegebenen Informationen.
7. Sollten sich behandlungsbedürftige Befunde ergeben, werden Sie von Ihrem behandelnden Arzt kontaktiert und **erhalten eine entsprechende Therapie**.

Klinikum rechts der Isar der Technischen Universität München
Innere Medizin II, Infektiologie, Studienzentrum

Figure S2. Manual for oropharyngeal sampling.

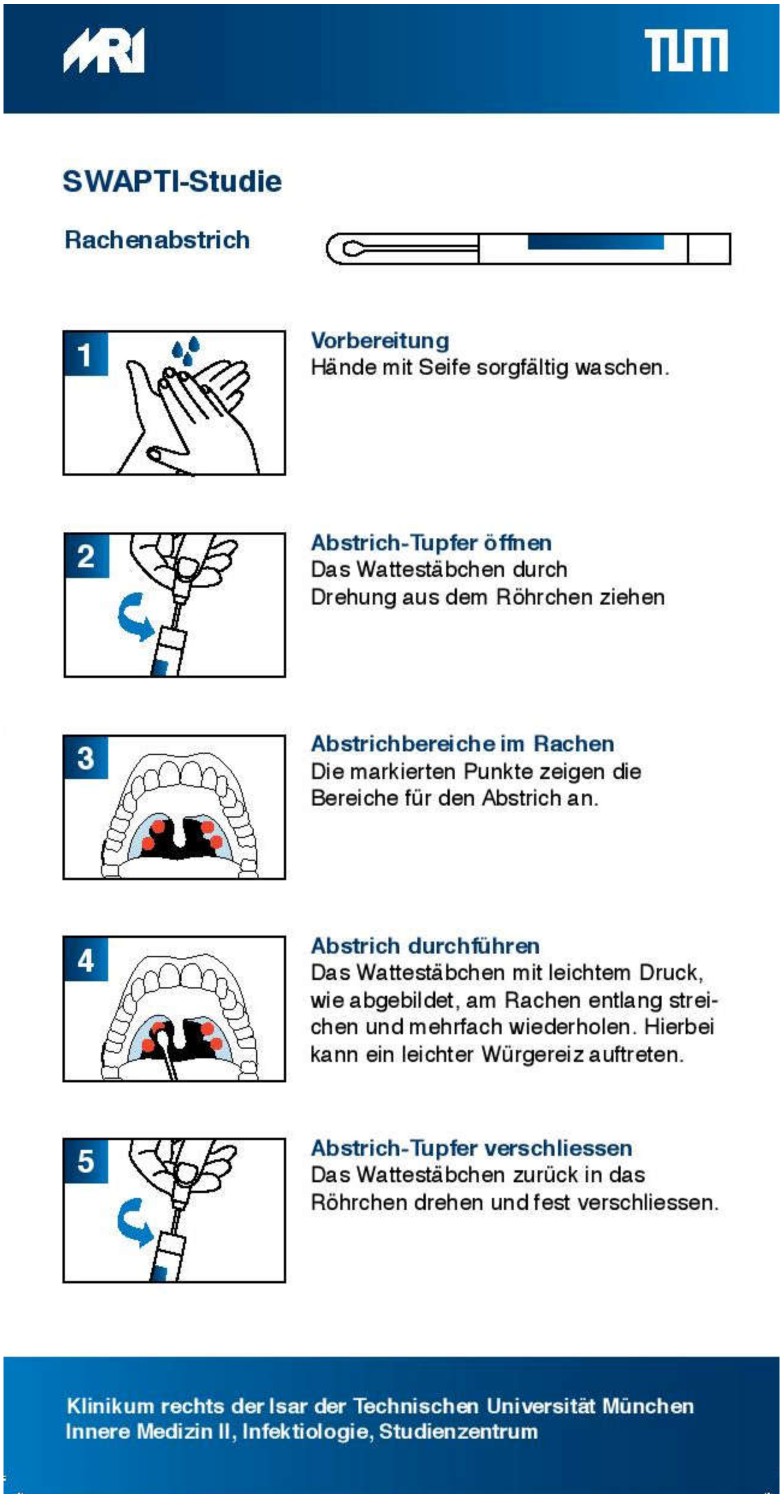

SWAPTI-Studie

Rachenabstrich

1 **Vorbereitung**
Hände mit Seife sorgfältig waschen.

2 **Abstrich-Tupfer öffnen**
Das Wattestäbchen durch Drehung aus dem Röhrchen ziehen

3 **Abstrichbereiche im Rachen**
Die markierten Punkte zeigen die Bereiche für den Abstrich an.

4 **Abstrich durchführen**
Das Wattestäbchen mit leichtem Druck, wie abgebildet, am Rachen entlang streichen und mehrfach wiederholen. Hierbei kann ein leichter Würgereiz auftreten.

5 **Abstrich-Tupfer verschliessen**
Das Wattestäbchen zurück in das Röhrchen drehen und fest verschliessen.

Klinikum rechts der Isar der Technischen Universität München
Innere Medizin II, Infektiologie, Studienzentrum

Figure S3. Manual for rectal sampling.

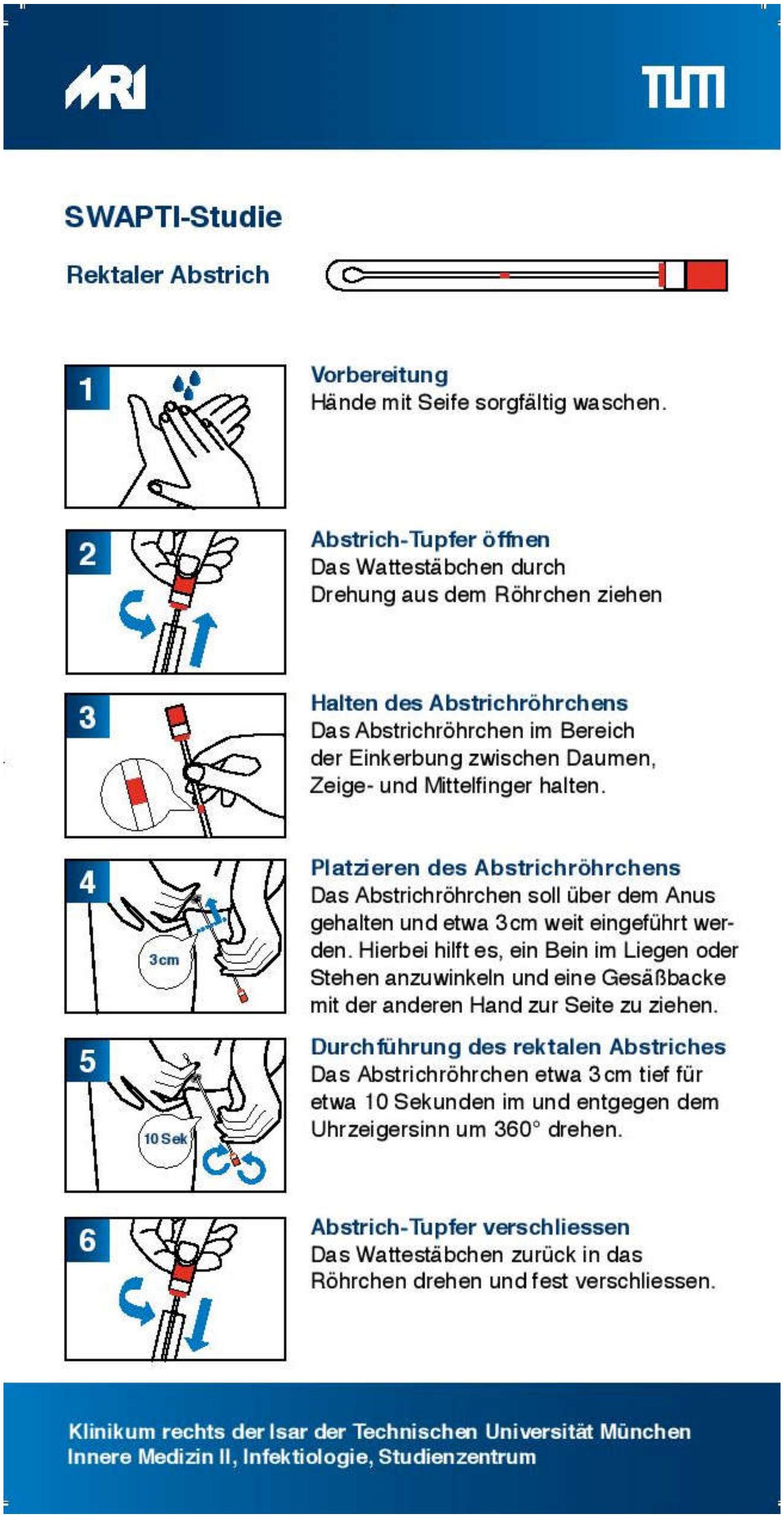

Figure S4. Manual for urine collection.

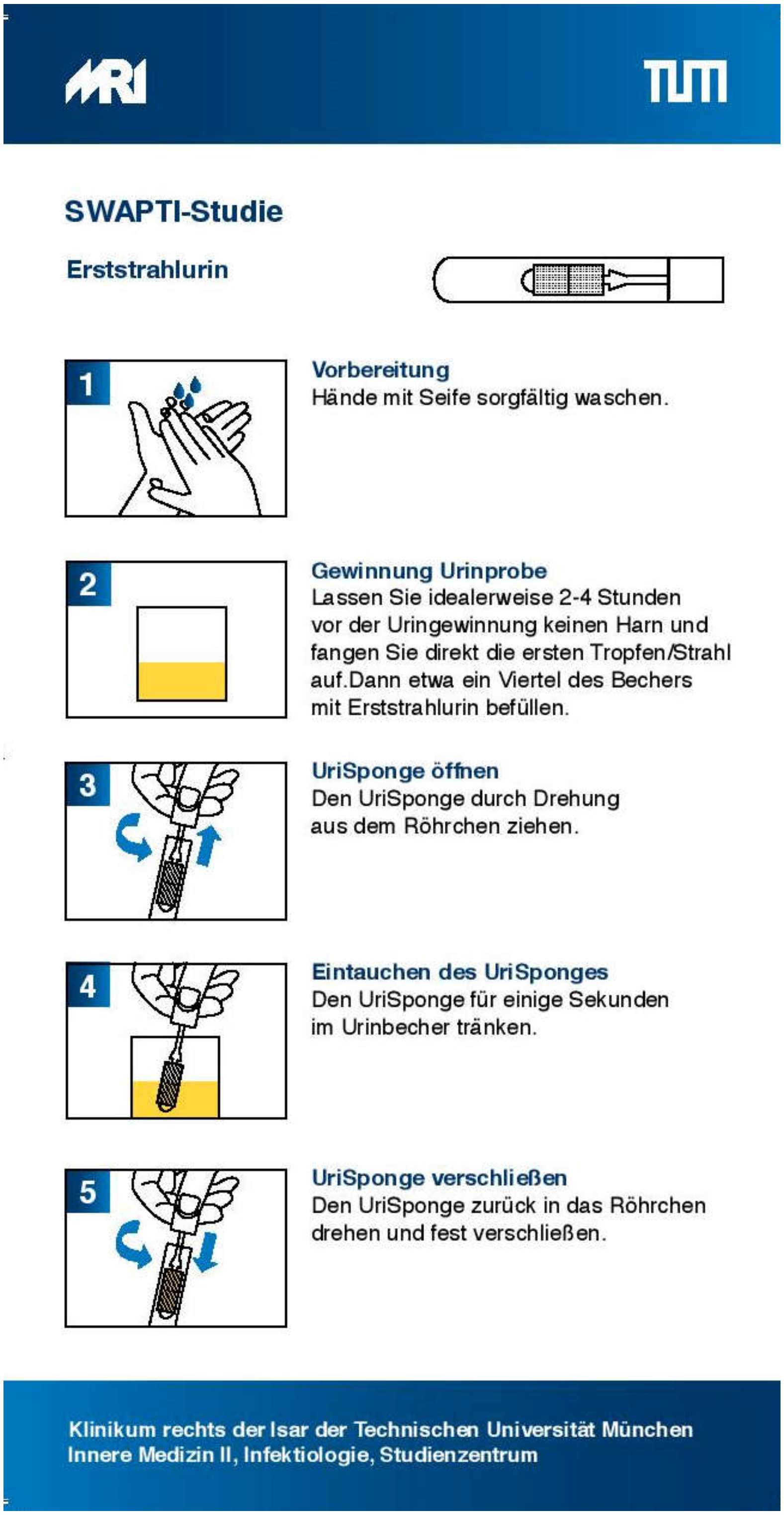

SWAPTI-Studie

Erststrahlurin

1 Vorbereitung
Hände mit Seife sorgfältig waschen.

2 Gewinnung Urinprobe
Lassen Sie idealerweise 2-4 Stunden vor der Uringewinnung keinen Harn und fangen Sie direkt die ersten Tropfen/Strahl auf.Dann etwa ein Viertel des Bechers mit Erststrahlurin befüllen.

3 UriSponge öffnen
Den UriSponge durch Drehung aus dem Röhrchen ziehen.

4 Eintauchen des UriSponges
Den UriSponge für einige Sekunden im Urinbecher tränken.

5 UriSponge verschließen
Den UriSponge zurück in das Röhrchen drehen und fest verschließen.

Klinikum rechts der Isar der Technischen Universität München
Innere Medizin II, Infektiologie, Studienzentrum
